# Supplementary material for: Modeling glioblastoma heterogeneity as a dynamic network of cell states
Source: Mol Syst Biol. 2021 Sep 16;17(9):e10105. doi: 10.15252/msb.202010105 (PMC8444284; doi:10.15252/msb.202010105)
Supplement: Supplementary file 6 — Source Data for Figure 5 [file MSB-17-e10105-s004.zip › Figure5A_sourcedata/GSEA_3071/hallmarks_stateII.GseaPreranked.1623742536692/HALLMARK_KRAS_SIGNALING_UP.html]

Details for gene set HALLMARK\_KRAS\_SIGNALING\_UP[GSEA]

|  || Dataset | state23071 |
| Phenotype | NoPhenotypeAvailable |
| Upregulated in class | na\_neg |
| GeneSet | HALLMARK\_KRAS\_SIGNALING\_UP |
| Enrichment Score (ES) | -0.38472554 |
| Normalized Enrichment Score (NES) | -1.9004953 |
| Nominal p-value | 0.0016835016 |
| FDR q-value | 0.061473813 |
| FWER p-Value | 0.072 |
Table: GSEA Results Summary

  

Fig 1: Enrichment plot: HALLMARK\_KRAS\_SIGNALING\_UP      
 Profile of the Running ES Score & Positions of GeneSet Members on the Rank Ordered List

  

| PROBE | GENE SYMBOL | GENE\_TITLE | RANK IN GENE LIST | RANK METRIC SCORE | RUNNING ES | CORE ENRICHMENT || 1 | LIF |  |  | 64 | 1.184 | 0.0183 | No |
| 2 | PLAUR |  |  | 185 | 0.893 | 0.0143 | No |
| 3 | INHBA |  |  | 196 | 0.876 | 0.0370 | No |
| 4 | AKAP12 |  |  | 349 | 0.711 | 0.0199 | No |
| 5 | ITGB2 |  |  | 476 | 0.639 | 0.0071 | No |
| 6 | ERO1A |  |  | 767 | 0.510 | -0.0500 | No |
| 7 | ITGBL1 |  |  | 773 | 0.507 | -0.0366 | No |
| 8 | F13A1 |  |  | 897 | 0.472 | -0.0535 | No |
| 9 | LCP1 |  |  | 950 | 0.459 | -0.0532 | No |
| 10 | HBEGF |  |  | 972 | 0.451 | -0.0454 | No |
| 11 | GFPT2 |  |  | 1022 | 0.437 | -0.0449 | No |
| 12 | TNFAIP3 |  |  | 1070 | 0.424 | -0.0444 | No |
| 13 | ITGA2 |  |  | 1114 | 0.415 | -0.0431 | No |
| 14 | FBXO4 |  |  | 1210 | 0.394 | -0.0552 | No |
| 15 | PPP1R15A |  |  | 1284 | 0.381 | -0.0623 | No |
| 16 | IL7R |  |  | 1360 | 0.366 | -0.0704 | No |
| 17 | NRP1 |  |  | 1372 | 0.362 | -0.0627 | No |
| 18 | EPHB2 |  |  | 1420 | 0.353 | -0.0641 | No |
| 19 | YRDC |  |  | 1492 | 0.342 | -0.0719 | No |
| 20 | PRELID3B |  |  | 1537 | 0.334 | -0.0732 | No |
| 21 | BIRC3 |  |  | 1626 | 0.320 | -0.0857 | No |
| 22 | GALNT3 |  |  | 1706 | 0.310 | -0.0963 | No |
| 23 | ETS1 |  |  | 1721 | 0.308 | -0.0910 | No |
| 24 | BPGM |  |  | 1832 | 0.294 | -0.1097 | No |
| 25 | EMP1 |  |  | 1987 | 0.274 | -0.1399 | No |
| 26 | DNMBP |  |  | 2112 | 0.262 | -0.1631 | No |
| 27 | PTPRR |  |  | 2178 | 0.254 | -0.1719 | No |
| 28 | AVL9 |  |  | 2256 | -0.255 | -0.1836 | No |
| 29 | SPRY2 |  |  | 2455 | -0.278 | -0.2246 | No |
| 30 | TMEM158 |  |  | 2476 | -0.280 | -0.2215 | No |
| 31 | ADAM17 |  |  | 2627 | -0.305 | -0.2498 | No |
| 32 | NIN |  |  | 2630 | -0.305 | -0.2415 | No |
| 33 | EVI5 |  |  | 2635 | -0.306 | -0.2337 | No |
| 34 | TSPAN13 |  |  | 2706 | -0.318 | -0.2419 | No |
| 35 | ZNF277 |  |  | 2733 | -0.323 | -0.2390 | No |
| 36 | HKDC1 |  |  | 2819 | -0.339 | -0.2503 | No |
| 37 | GLRX |  |  | 2834 | -0.342 | -0.2439 | No |
| 38 | LY96 |  |  | 2950 | -0.364 | -0.2619 | No |
| 39 | CPE |  |  | 2994 | -0.371 | -0.2618 | No |
| 40 | SOX9 |  |  | 3230 | -0.435 | -0.3075 | No |
| 41 | HDAC9 |  |  | 3288 | -0.453 | -0.3085 | No |
| 42 | G0S2 |  |  | 3545 | -0.568 | -0.3555 | No |
| 43 | ID2 |  |  | 3664 | -0.654 | -0.3659 | Yes |
| 44 | GYPC |  |  | 3722 | -0.701 | -0.3598 | Yes |
| 45 | CXCR4 |  |  | 3733 | -0.710 | -0.3418 | Yes |
| 46 | PRRX1 |  |  | 3758 | -0.737 | -0.3265 | Yes |
| 47 | PLAT |  |  | 3766 | -0.752 | -0.3066 | Yes |
| 48 | SATB1 |  |  | 3776 | -0.767 | -0.2868 | Yes |
| 49 | KIF5C |  |  | 3813 | -0.800 | -0.2726 | Yes |
| 50 | DUSP6 |  |  | 3899 | -0.946 | -0.2664 | Yes |
| 51 | ETV1 |  |  | 3934 | -1.026 | -0.2453 | Yes |
| 52 | GPRC5B |  |  | 3937 | -1.032 | -0.2161 | Yes |
| 53 | TRIB2 |  |  | 3944 | -1.041 | -0.1876 | Yes |
| 54 | GNG11 |  |  | 3952 | -1.062 | -0.1587 | Yes |
| 55 | ETV5 |  |  | 3971 | -1.116 | -0.1311 | Yes |
| 56 | PCSK1N |  |  | 3987 | -1.179 | -0.1008 | Yes |
| 57 | RGS16 |  |  | 3996 | -1.227 | -0.0675 | Yes |
| 58 | MAP3K1 |  |  | 4032 | -1.477 | -0.0336 | Yes |
| 59 | IGFBP3 |  |  | 4058 | -1.726 | 0.0099 | Yes |
Table: GSEA details [plain text format]

  

Fig 2: HALLMARK\_KRAS\_SIGNALING\_UP: Random ES distribution      
 Gene set null distribution of ES for **HALLMARK\_KRAS\_SIGNALING\_UP**
